# Drug therapy for hereditary cancers

**DOI:** 10.1186/1897-4287-9-5

**Published:** 2011-08-06

**Authors:** Evgeny N Imyanitov, Vladimir M Moiseyenko

**Affiliations:** 1 Laboratory of Molecular Oncology, N.N. Petrov Institute of Oncology, St.-Petersburg, 197758, Russia; 2 Department of Oncology, St.-Petersburg Medical Academy for Postgraduate Studies, St.-Petersburg, 191015, Russia; 3 Department of Medical Genetics, St.-Petersburg Pediatric Medical Academy, St.-Petersburg, 194100, Russia

## Abstract

Tumors arising in patients with hereditary cancer syndromes may have distinct drug sensitivity as compared to their sporadic counterparts. Breast and ovarian neoplasms from *BRCA1 *or *BRCA2 *mutation carriers are characterized by deficient homologous recombination (HR) of DNA, that makes them particularly sensitive to platinum compounds or inhibitors of poly (ADP-ribose) polymerase (PARP). Outstandingly durable complete responses to high dose chemotherapy have been observed in several cases of *BRCA*-related metastatic breast cancer (BC). Multiple lines of evidence indicate that women with *BRCA1*-related BC may derive less benefit from taxane-based treatment than other categories of BC patients. There is virtually no reports directly assessing drug response in hereditary colorectal cancer (CRC) patients; studies involving non-selected (i.e., both sporadic and hereditary) CRC with high-level microsatellite instability (MSI-H) suggest therapeutic advantage of irinotecan. Celecoxib has been approved for the treatment of familial adenomatous polyposis (FAP). Hereditary medullary thyroid cancers (MTC) have been shown to be highly responsive to a multitargeted tyrosine kinase inhibitor vandetanib, which exerts specific activity towards mutated RET receptor. Given the rapidly improving accessibility of DNA analysis, it is foreseen that the potential predictive value of cancer-associated germ-line mutations will be increasingly considered in the future studies.

## Introduction

Search for hereditary cancer genes was always regarded as a high priority translational research with immediate health impact. It was foreseen, that the discovery of tumor predisposing mutations and the development of appropriate genetic tests will allow identifying yet healthy subjects, who are at nearly fatal risk of specific type of cancer and thus may benefit from a timely medical intervention. Since the discovery of major cancer genes in the mid 1990s, thousands of mutation carriers have been subjected to intensive surveillance programs in order to secure early diagnosis of the disease [1]. The preventive surgery has been applied in some instances, that led to a proven reduction of cancer-specific mortality [2]. While the initial practical focus of cancer genetic research was limited to various aspects of cancer detection and prevention, it is now getting increasingly recognized that hereditary tumors may have distinct bioclinical characteristics and thus require tailored treatment strategies.

### Breast-ovarian cancer syndrome

The best-known hereditary cancer genes, *BRCA1 *and *BRCA2*, contribute to substantial share of breast and ovarian tumor incidence around the globe, and have been studied with significant level of comprehension [3]. Mutations in other relevant genes, such as *CHEK2, PALB2 (FANCN), ATM, NBN (NBS1), BRIP1 (FANCJ, BACH1)*, *BLM*, are less frequent and have not been subjected yet to systematic clinical studies.

#### BRCA1 and BRCA2

*BRCA1 *and *BRCA2 *genes play a central role in the repair of double-strand DNA breaks by homologous recombination (HR). Cancers in *BRCA *heterozygous individuals arise due to somatic inactivation of the remaining wild-type allele of the gene. This provides a critical biological difference between cancerous and normal cells: while *BRCA*-driven tumors are characterized by HR deficiency, normal tissues from the same individuals retain non-affected *BRCA *allele and the ability to cope with DNA damage [4,5]. Although *BRCA1*- and *BRCA2*-related cancers demonstrate somewhat distinct picture of genetic abnormalities [6,7], they both have increased number of gross chromosomal aberrations and therefore higher tumor grade [8-11]. Cells carrying multiple genetic lesions due to HR defect are normally eliminated by p53-guided defense mechanisms; there are convincing evidence from both human studies and murine models that *p53 *inactivation is an absolute prerequisite for the propagation of *BRCA*-dysfunctional tumors cells [12-15].

*BRCA1 *has a wider spectrum of functions than *BRCA2 *[16,17]. In addition to DNA repair, *BRCA1 *is involved in breast cell differentiation and transcriptional regulation of the estrogen receptor (ER) [18]. It has been repeatedly shown, that the majority of *BRCA1*-mutated breast carcinomas (BC) do not express ER, while the hormonal receptor pattern in *BRCA2*-associated BC is similar to sporadic cases [19]. *BRCA1 *is also essential for the mitotic spindle checkpoint as it triggers cellular suicide in response to microtubule damage [20].

#### Preclinical studies

A large number of preclinical studies aimed to assess specific sensitivity of *BRCA1*- and *BRCA2*-defective cells to various anticancer agents. Surprisingly, while the effect of individual compounds has been repeatedly evaluated in diverse model systems (Table 1), there was virtually no attempt to compare clinically relevant combinations of the drugs (Table 2). This may constitute a critical gap between preclinical and clinical research, as single-agent therapy is almost never used as initial treatment of breast or ovarian cancers. It is highly likely, that the standard combinations of cytotoxic compounds produce distinct spectrum of DNA lesions and therefore mediate distinct responses of *BRCA*-deficient cells when compared to the same drugs acting alone.

**Table 1:** Drug sensitivity of breast-ovarian cancer syndrome related tumors: preclinical evidence

Study	Study design and main findings
*BRCA1*	
Husain et al. [21]	Antisense inhibition of *BRCA1 *expression in the cisplatin-resistant clone of SKOV3 ovarian cancer cell line restored sensitivity to the drug.

Bhattacharyya et al. [22]	*BRCA1*-deficient mouse embryonic stem cells were more sensitive to cisplatin than isogenic *BRCA1*-proficient cells, as determined by a clonogenic assay.

Brodie et al. [45]	Cell lines, which were generated from mammary tumors growing in genetically engineered *BRCA1*-deficient mice, demonstrated high sensitivity to doxorubicin in a cell survival assay.

Lafarge et al. [23]	Inhibition of *BRCA1 *expression in HBL100 breast cancer cells led to increased sensitivity to cisplatin and etoposide, but resistance to paclitaxel and vincristine, as assessed by the rhodamine B proliferation test.

Moynahan et al. [42]	Increased sensitivity of *BRCA1*-deficient mouse embryonic stem cells to mitomycin C in a clonogenic assay; reversed upon correction of *BRCA1*-mutated allele by gene targeting.

Mullan et al. [49]	Tetracycline regulated inducible expression of *BRCA1 *in MBR62-bcl2 breast cancer cell line increased sensitivity to paclitaxel and vincristine, by did not affect the response to cisplatin, doxorubicin, cyclophosphamide, 5-fluorouracil, or bleomycin, as determined by a clonogenic assay.

Fedier et al. [24]	Increased sensitivity of *BRCA1*-deficient mouse embryonic cells to the antiproliferative effect of camptothecin, topotecan, doxorubicin, mitoxantrone, etoposide, carboplatin, oxaliplatin, but not of 5-fluoroucil, gemcitabine, paclitaxel, docetaxel; increased apoptosis in response to doxorubicin but not to docetaxel.

Quinn et al. [25]	*BRCA1*-deficiency is associated in increased sensitivity to apoptosis caused by etoposide, bleomycin or cisplatin, but resistance to apoptotic response to paclitaxel or vinorelbine.

Tassone et al. [26]	*BRCA1*-mutant HCC1937 breast cancer cells were more sensitive to cisplatin, but less sensitive to doxorubicin and paclitaxel, than *BRCA1*-proficient MCF7 and MDA-MB-231 cells, as determined by the MTT test. Transfection of the wild-type *BRCA1 *in HCC1937 cells decreased their sensitivity to cisplatin, but restored sensitivity to doxorubicin and paclitaxel; this effect was at least in part attributed to the modulation of the apoptotic response.

Zhou et al. [52]	SNU251 ovarian cancer cell line carrying truncation of 49 C-terminal aminoacids of the *BRCA1 *gene (partial deletion of 2^nd ^BRCT domain) demonstrated increased sensitivity to paclitaxel in a cell viability assay; this effect was reversed by the introduction of the wild-type *BRCA1*.

Farmer et al. [57]	siRNA directed or chemical inhibition of PARP profoundly inhibited clonogenicity of *BRCA1*-deficient mouse embryonic stem cells as compared to *BRCA1*-proficient isogenic cell lines; similar results were obtained upon simultaneous inhibition of *BRCA1 *and PARP in MCF7 breast cancer cell line. This effect was attributed to the massive growth arrest and subsequent apoptosis.

Tassone et al. [53]	*BRCA1*-mutant HCC1937 breast cancer cells were more sensitive to vinorelbine than *BRCA1*-proficient MCF7 and MDA-MB-468 cells; when docetaxel was used, HCC1937 were similarly sensitive as compared to MCF7, and less sensitive than MDA-MB-468 (MTT test). Transfection of the wild-type *BRCA1 *in HCC1937 cells rendered resistance to vinorelbine, but slightly increased sensitivity to docetaxel. The effect of vinorelbine was at least in part attributed to the modulation of the apoptotic response.

Yun et al. [44]	*BRCA1*-deficient mouse embryonic fibroblasts were significantly more sensitive to mitomycin C than *BRCA1*-wild-type expressing isogenic cells, as demonstrated by a clonogenic assay. The effect of mitomycin C is mediated through massive S-phase arrest followed by apoptosis.

Bartz et al. [29]	*BRCA1 *inhibition strongly increased sensitivity of HeLa cells to cisplatin, as revealed by siRNA screen.

Chabalier et al. [50]	siRNA-directed inactivation of *BRCA1 *function in MCF7 breast cancer cells rendered resistance to paclitaxel-induced growth inhibition and mitotic arrest.

Xing and Orsulic [30]	*BRCA1*-deficient transformed mouse ovarian surface epithelial cell lines demonstrated higher sensitivity to cisplatin as compared to *BRCA1*-proficient isogenic cells, while no differential sensitivity to paclitaxel was observed.

Donawho et al. [31]	Veliparib potentiated inhibitory effect of cisplatin, carboplatin and cyclophosphamide towards human *BRCA1*-mutated breast cancer xenografts (MX-1) growing in immunocompromised mice.

Rottenberg et al. [32]	Doxorubicin, docetaxel and cisplatin inhibited growth of mammary tumors in genetically engineered *BRCA1*-deficient mice. Treated tumors eventually acquired resistance to doxorubicin and docetaxel, but not to cisplatin.

Treszezamsky et al. [48]	*BRCA1*-mutant HCC1937 breast cancer cells showed increased sensitivity to etoposide as compared to *BRCA1*-wild-type expressing isogenic cells, as shown by a clonogenic assay.

Yamane et al. [117]	*BRCA1*-mutant HCC1937 breast cancer cell line showed increased survival and decreased apoptosis in response 6-thioguanine as compared to *BRCA1*-wild-type expressing isogenic cells.

Shafee et al. [34]	Cisplatin caused marked regression of *BRCA1*-deficient tumors growing in genetically engineered mice, while doxorubicin exerted only marginal effect.

Rottenberg et al. [59]	Olaparib inhibited growth of mammary tumors in genetically engineered *BRCA1*-deficient mice. Combination of olaparib with cisplatin or carboplatin produced longer recurrence-free and overall survival than olaparib alone.

Promkan et al. [51]	shRNA-directed inhibition of *BRCA1 *expression in MCF7 and MDA-MB-231 breast cancer cells decreased cytotoxicity of paclitaxel. Breast cancer cell lines carrying *BRCA1 *mutation (HCC1937 and MDA-MB-436) also demonstrated low sensitivity to paclitaxel.

Santarosa et al. [36]	Antisense inhibition of *BRCA1 *expression in HBL100, MCF7 and T47D breast cancer cells led to increased sensitivity to mitomycin C and cisplatin, but not to doxorubicin and etoposide, as determined by a clonogenic assay. Similar results were obtained on the *BRCA1*-mutated HCC1937 breast cancer cell line. This effect was attributed to premature senescence of the chemosensitive cells.

Tassone et al. [37]	Cisplatin induced almost complete growth inhibition of HCC1937-derived (*BRCA1*-mutated) breast cancer xenografts, while *BRCA1*-reconsituted HCC1937 xenografted tumors showed only partial response to cisplatin treatment.

Zander et al. [54]	Topotecan inhibited growth of mammary tumors in genetically engineered *BRCA1*-deficient mice.

Drew et al. [39]	AG014699 (PARP inhibitor) was highly cytotoxic against breast cancer cells with mutationally inactivated (MDA-MB-436) or epigenetically silenced (UACC3199) *BRCA1*, as determined by a clonogenic assay. *BRCA1*-deficient HCC1937 cells were more sensitive to AG014699 than the isogenic cell line with restored *BRCA1 *function (rhodamine B proliferation test). AG014699 and carboplatin efficiently inhibited growth of MDA-MB-436 and UACC3199 derived xenografted tumors.

Goldberg et al. [60]	Nanoparticle-mediated delivery of PARP1-specific siRNA inhibited growth of *BRCA1*-deficient mouse ovarian cancer allografts; this effect was at least in part attributed to apoptotic death of targeted cells.

***BRCA2***	

Abbott et al. [47]	Increased sensitivity of *BRCA2*-deficient pancreatic cancer cell line CAPAN1 to mitoxantrone, etoposide and amsacrine, but not to paclitaxel, as assessed by a cell survival assay. Antisense down-regulation of *BRCA2 *in *BRCA2*-proficient pancreatic cancer cells resulted in hypersensitivity to mitoxantrone. CAPAN1 xenografted tumors showed nearly complete response to mitoxantrone and marked response to etoposide.

Yu et al. [41]	Increased sensitivity of *BRCA2*-deficient vs. *BRCA2*-wild-type mouse lymphocytes to the mitomycin C, as determined by a cell survival assay.

Rahden-Staron et al, [55]	Increased sensitivity of *BRCA2*-deficient Chinese hamster VS8 fibroblasts to camptothecin in a cell survival assay.

Samouelian et al. [27]	Increased sensitivity of *BRCA2*-deficient ovarian cancer cell line TOV81 to cisplatin, but not to camptothecin or paclitaxel, as assessed by a cell survival assay.

van der Heijden et al. [28]	Increased sensitivity of *BRCA2*-deficient pancreatic cancer cell line CAPAN1 to cisplatin and mitomycin C, as shown by a cell survival assay. Increased sensitivity to mitomycin C induced G2/M cell cycle growth arrest.

Bryant et al. [56]	Chemical inhibition of PARP profoundly inhibited clonogenicity of *BRCA2*-deficient Chinese hamster VS8 fibroblasts as compared to parental V79 cells. Similar results were obtained upon simultaneous inhibition of *BRCA2 *and PARP in MCF7 and MDA-MB-231 breast cancer cell lines.

Farmer et al. [57]	siRNA directed or chemical inhibition of PARP profoundly inhibited clonogenicity of *BRCA2*-deficient mouse embryonic stem cells as compared to *BRCA2*-proficient isogenic cell lines; similar results were obtained on *BRCA2*-deficient Chinese hamster ovarian cancer cell line. This effect was attributed to the massive growth arrest and subsequent apoptosis. Chemical PARP inhibitor suppressed growth of *BRCA2*-deficient xenografts in athymic mice.

Gallmeier and Kern [155], McCabe et al. [58]	*BRCA2*-deficient CAPAN1 pancreatic cancer cells were sensitive to highly active PARP inhibitors, while moderately active PARP inhibitors did not affect cell survival.

van der Heijden et al. [43]	*BRCA2*-deficient CAPAN1 xenografted tumors showed marked response to mitomycin C.

Bartz et al. [29]	*BRCA2 *inhibition strongly increased sensitivity of HeLa cells to cisplatin, as revealed by siRNA screen.

Treszezamsky et al. [48]	*BRCA2*-deficient EUFA423 fibroblasts showed increased sensitivity to etoposide as compared to *BRCA1*-wild-type expressing isogenic cells, as shown by a clonogenic assay.

Evers et al. [33]	Olaparib, cisplatin, mitomycin C and temozolomide effectively inhibited growth of *BRCA2*-deficent vs. *BRCA2*-proficient mouse cell lines, while doxorubicin, docetaxel and 5-fluorouracil showed no difference. Synergism between olaparib and cisplatin was observed for *BRCA2*-deficient but not for *BRCA2*-proficient cells.

Hay et al. [35]	*BRCA2*-deficient mammary tumors growing in genetically engineered mice demonstrated high sensitivity to olaparib and carboplatin.

Evers et al. [38]	High-throughput pharmaceutical screen involving *BRCA2*-deficent vs. *BRCA2*-proficient mouse mammary tumor cell lines identified alkylating agents (chlorambucil, melphalane, nimustine) as the most potent and specific inhibitors of cell growth; differential inhibition was also registered for carboplatin, camptothecin and ellipticine. *BRCA2*-deficient mammary tumors, either transplanted or growing in genetically engineered mice, demonstrated high sensitivity to alkylating compounds. Synergistic interaction between alkylators and olaparib was observed both in vitro and in vivo.

Issaeva et al. [46]	Chemical library screen identified 6-thioguanine as the most potent inhibitor of the survival of *BRCA2*-deficient human sarcoma U2OS cells and Chinese hamster VS8 fibroblasts. High efficacy of both 6-thioguanine and AG014699 (PARP inhibitor) against *BRCA2*-deficient xenografted tumors. Comparison of *BRCA2*-deficient VS8 cells versus isogenic *BRCA2*-expressing VS8+B2 cells: higher sensitivity to temozolomide, camptothecin and doxorubicin, but lower sensitivity to gemcitabine and paclitaxel.

Drew et al. [39]	AG014699 (PARP inhibitor) was highly cytotoxic against *BRCA2*-deficient CAPAN1 pancreatic cancer cells, as determined by a clonogenic assay. AG014699 and carboplatin efficiently inhibited growth of CAPAN1-derived tumor xenografts, with the most pronounced effect while using combination of these drugs.

Kortmann et al. [40]	*BRCA2*-deficient ovarian cancer xenografts showed marked response to olaparib, carboplatin, and olaparib plus carboplatin, whereas *BRCA2*-proficient xenografts responded only to carboplatin and olaparib plus carboplatin.

***PALB2***	

Villarroel et al. [97]	High sensitivity of *PALB2*-deficient xenografted pancreatic tumor to mitomycin C and cisplatin but not to gemcitabine.

**Other genes**	

McCabe et al. [93]	RNA-interference driven inhibition of *NBN (NBS1)*, *CHEK2 *or some other genes involved in homologous recombination increased sensitivity of cultured cells to PARP inhibitors, as determined by cell survival assays.

Bartz et al. [29]	*BRIP1 *inhibition strongly increased sensitivity of HeLa cells to cisplatin, as revealed by siRNA screen.

**Table 2:** Drug sensitivity of breast-ovarian cancer syndrome related tumors: clinical evidence

Study	Study design and main findings^1^
**Breast cancer**	

Kloos et al. [156]; Delagoge et al. [65]	15 *BRCA1 *carriers, 5 *BRCA2 *carriers and 57 matched controls were treated by anthracycline-based neoadjuvant therapy. Objective responses in 100%, 80%, 63% and pCR in 53%, 0% and 14%, respectively.

Chappuis et al. [66]; Wong Wong Keet et al. [71]	7 *BRCA1 *and 4 *BRCA2 *carriers were treated by 3-4 cycles of anthracycline-based neoadjuvant therapy, 10 (91%) of them achieved cCR; pCR were documented in 4 (44%) out of 9 evaluable cases. 27 patients served as controls: cCR and pCR were detected in 8 (30%) and 1 (4%) patients, respectively. These patients were followed for a median period of 7 years. Among complete clinical responders, only 1 (17%) out 6 *BRCA1 *carriers but 3 (75%) out of 4 *BRCA2 *carriers died of breast cancer.

Warner et al. [67]	Rapid radiological disappearance and complete pathological response in a *BRCA1 *carrier treated by neoadjuvant FEC.

Petit et al. [72]	55 triple-negative BC treated by neoadjuvant FEC. The subgroup of *BRCA1 *carriers showed lower pCR rate (2/12, 17%) than overall (23/55, 42%).

Chrisanthar et al. [94]	2/3 (67%) *CHEK2 *mutation carriers as compared to 8/104 (8%) non-carriers progressed on neoadjuvant epirubicin monotherapy for locally advanced breast cancer.

Hubert et al. [68]	15 *BRCA1 *and 7 *BRCA2 *stage III breast cancers were treated by anthracycline-based neoadjuvant therapy. cCR in 6/15 (40%) and 1/7 (14%), pCR in 2/15 (13%) and 0 patients, respectively.

Melichar et al. [157]	Case report on 2 related *BRCA1 *carriers, whose tumor demonstrated pCR upon dose dense AC and sequential weekly paclitaxel.

Wysocki et al. [75]	19 non-responders to docetaxel have been analyzed; 5 (26%) of them turned out to be *BRCA1 *carriers.

Fong et al. [102]	Phase I study dose escalation study for olaparib. 3 *BRCA2*-related chemotherapy refractory breast cancer patients were evaluable for the treatment efficacy; 1 OR and 1 SD were observed. No responses in *BRCA*-mutation-negative cases included in the study.

Fourquet et al. [69]	Higher rate of cCR to anthracycline-based neoadjuvant therapy in *BRCA1*/2-carriers (15/33, 46%) vs. non carriers (7/41, 17%)

Huang et al. [80]	Case report: metastatic breast cancer in a *BRCA2*-carrier was treated by several regimens of high dose therapy (epirubicin, alkylating agents, cisplatin, other cytotoxic drugs); CR with duration of 11+ years was observed.

Kriege et al. [73]	93 *BRCA1 *and 28 *BRCA2 *carriers received 1^st^-line chemotherapy (mainly anthracycline-based or CMF (CMF-like)) for the treatment of metastatic disease. Sporadic cases (n = 121) were used as a control. Marginally improved outcomes in *BRCA1 *carriers: OR: 66% vs. 50%; median PFS: 7.6 vs. 6.7 months; median OS: 15.0 vs. 13.6 months; significantly improved outcomes in *BRCA2 *carriers: OR: 89% vs. 50%; median PFS: 11.4 vs. 6.7 months; median OS: 19.3 vs. 13.6 months.

Rhiem et al. [158]	Case report: major response of heavily pretreated metastatic *BRCA1*-related cancer to the combination of cisplatin and gemcitabine, with the duration > 6 months.

Vollebergh et al. [81]	Long term outcome of high dose therapy (carboplatin, thiotepa and cyclophosphamide) is analyzed in 40 patients with metastatic breast cancer. 6 patients remained on complete remission at the time of the analysis (56+ - 150+ months); all these 6 patients demonstrated chromosomal imbalances characteristic for *BRCA1*-related cancers. Complete long term responders included 1 out of 2 *BRCA1*- and 1 out of 2 *BRCA2*-carriers.

Byrski et al. [70,159,160]	102 *BRCA1 *carriers treated by neoadjuvant chemotherapy. pCR in 1/14 (7%) women receiving CMF, 2/25 (8%) patients exposed to doxorubicin and docetaxel, 11/51 (22%) cases treated by AC or FAC, 10/12 (83%) women receiving cisplatin.

Moiseyenko et al. [74]	Case-report: lack of response of a chemonaive *BRCA1*-related cancer to the 1^st ^line epirubicin-docetaxel combination, than major response to the 2^nd ^line single-agent cisplatin.

Silver et al. [78]	28 stage II or III triple-negative breast cancers were treated by 4 cycles of neoadjuvant cisplatin monotherapy. Two *BRCA1*-carriers were included in the study, and both achieved pCR.

Kriege et al. [76]	32 *BRCA1 *carriers, 13 *BRCA2 *carriers and 95 controls treated by taxane monotherapy or taxane-trastuzumab combination. Inferior results in *BRCA1 *carriers (OR rate = 23% vs. 38%; PD in 60% vs. 19%; median PFS = 2.2 vs. 4.9 months); this difference retained significance only in hormone receptor-negative cases, while hormone receptor-positive tumors showed similar response rates and PFS. *BRCA2 *carriers: higher OR rate (89% vs. 38%), similar median PFS (7.1 vs. 5.7 months).

Tutt et al. [82]	*BRCA1*- and *BRCA2*-related metastatic breast cancers, with at least 1 prior chemotherapy regimen, treated by high-dose (n = 27) or low-dose (n = 27) olaparib. OR in 41% and 22%; SD in 44% and 44%; median PFS = 5.7 and 3.8 months.

Sokolenko et al. [104]	5 *BLM*-related breast cancers treated by conventional neoadjuvant chemotherapy; 3 patients showed nearly complete pathologic response, and 2 remaining women demonstrated partial response.

**Ovarian cancer**	

Cass et al. [83]	34 *BRCA *carriers (22 *BRCA1 *and 12 *BRCA2*) vs. 37 non-carriers were included in the study; 29 vs. 25 had stage III-IV disease and were therefore considered for the analysis of treatment outcome; 22 vs. 18 received paclitaxel plus carboplatin, and 7 vs. 7 were treated by cyclophosphamide plus carboplatin. *BRCA1*/2 carriers demonstrated higher OR rate (21/24 (88%) vs. 9/19 (47%)) and longer median OS (91 vs. 54 months) than non-carriers.

Tan et al. [84]	Therapy response was compared in 22 *BRCA *carriers (17 *BRCA1 *and 5 *BRCA2*) vs. 44 non carriers matched by stage, histological subtype, age at diagnosis and year at diagnosis. Higher sensitivity to platinum-based therapy in the 1^st ^line (OR: 96% vs. 59%; CR: 82% vs. 43%), 2^nd ^line (92% vs. 41%) and 3^rd ^line treatment (100% vs. 14%); longer median OS (8.4 vs. 2.9 years).

Fong et al. [102]	Phase I study dose escalation study for olaparib. 15 *BRCA*-related (14 *BRCA1 *and 1 *BRCA2*) chemotherapy refractory ovarian cancer patients were evaluable for treatment efficacy; 8 (53%) OR and 1 (7%) SD were observed. No responses in *BRCA*-mutation-negative cases included in the study.

Leunen et al. [161]	6 patients with relapsed *BRCA*-related ovarian cancer, treated by multiple lines of therapy including dose-dense paclitaxel-carboplatin; all patients responded to the therapy; median OS = 37 months as compared to 18 months in the historical control.

Melichar et al. [86]	Case report on a *BRCA1*-related ovarian cancer relapsed after prolonged post-surgical paclitaxel-carboplatin therapy; each of 4 consequent relapses showed complete response to paclitaxel-cisplatin combination.

Audeh et al. [87]	*BRCA1*- and *BRCA2*-related recurrent ovarian cancers, with at least 1 prior chemotherapy regimen, treated by high-dose (n = 33) or low-dose (n = 24) olaparib. OR in 33% and 15%; SD in 36% and 29%; median duration of response = 290 and 269 days.

Fong et al. [88]	Phase I dose escalation and single-stage expansion trial for olaparib, given to 50 patients (including 41 *BRCA1 *and 7 *BRCA2 *carriers) with advanced ovarian cancer, previously treated by platinum based therapy. OR in 20 (40%) and SD in 3 (6%) patients. Median duration of response = 28 weeks. Strong correlation with platinum sensitivity of the disease.

Moiseyenko et al. [74]	Complete clinical response and nearly-complete pathological response of bulky tumor treated by 5 cycles of single-agent cisplatin.

Vencken et al. [85]	93 *BRCA1*-related, 13 *BRCA2*-related and 222 sporadic cancers analyzed for response to the 1^st ^line chemotherapy. CR or no evidence for disease: 87%, 92% and 71%, respectively; PD: 2%, 0% and 15%; median PFS: 2.1, 5.6 and 1.3 years; median OS: 5.9, > 10 and 2.9 years. Similar response rates in patients receiving combination of platinum and paclitaxel vs. those treated by platinum-based therapy without paclitaxel.

**Pancreatic cancer**	

Chalasani et al. [98]	Case report on a *BRCA2*-related pancreatic adenocarcinoma. 1^st ^line therapy included combination of gemcitabine with experimental alkylating agent, and resulted in a major tumor response. Then alkylating agent was discontinued due to toxicity, and PD was observed on single-agent gemcitabine. Experimental antiangiogenic drug was given in the 2^nd ^line without any effect. 3^rd ^line therapy included mitomycin C plus capecitabine, and again led to a major tumor response.

James et al. [99]	Case report on a *BRCA2*-related, *KRAS *wild-type pancreatic adenocarcinoma. Prolonged SD (56+ months) upon multiple lines of chemotherapy (combination of docetaxel, capecitabine and gemcitabine; then irinotecan monotherapy; then irinotecan plus cetuximab; then mitomycin C plus oxaliplatin; then mitomycin C plus irinotecan).

Villarroel et al. [97]	Case report on a *PALB2*-related pancreatic adenocarcinoma, which progressed under 1^st ^line single-agent gemcitabine, but showed major response after mitomycin C administration. Later mitomycin C was replaced by cisplatin due to toxicity of the former. The patient remains asymptomatic for 36+ months.

Fogelman et al. [100]	Case report on a *BRCA2*-related relapsed pancreatic adenocarcinoma, which rapidly regressed upon the combination of gemcitabine and iniparib. Subsequent surgery revealed a complete pathologic response.

**Other cancers**	

Fong et al. [102]	Phase I study dose escalation study for olaparib. 1 *BRCA2*-related castration-resistant prostate cancer patient was included in the study; he demonstrated significant, durable marker response as well as resolution of bone metastases. No responses in *BRCA*-mutation-negative cases.

Moule et al. [101]	Case report: complete response lasting for 10+ years in a *BRCA2 *carrier, whose advanced non-small cell lung cancer was treated by the combination of mitomycin C, cisplatin and vincristine.

Vesprini et al. [103]	Case report describing a patient with metastatic *BRCA2*-related prostate cancer, who was treated by cisplatin after becoming insensitive to androgen ablation. Cisplatin therapy resulted in normalization of prostate-specific antigen level and symptomatic relief for period of 8 months; docetaxel was administered after the disease progression, and also led to an evident tumor response.

^1^Drug combinations: CMF: cyclophosphamide, methotrexate and fluorouracil; AC: doxorubicin and cyclophosphamide; FAC: 5-fluorouracil, doxorubicin and cyclophosphamide; FEC: 5-fluorouracil, epirubicin and cyclophosphamide.

Treatment outcomes: OR: objective response; PR: partial response; CR: complete response; cCR: clinical complete response; pCR: pathological complete response; SD: stable disease; PD: progressive disease; PFS: progression-free survival; OS: overall survival.

There is an excellent consistency in the literature regarding high sensitivity of both *BRCA1*- and *BRCA2*-deficient cells to cisplatin and other platinum derivatives [21-40]. It is believed that the DNA crosslinks caused by platinating agents ultimately require homologous recombination to correctly repair DNA damage, so the *BRCA*-inactive cells cannot cope with this class of drugs. Similar consistency has been observed for another DNA-crosslinking agent, mitomycin C [28,33,36,41-44].

Controversial data have been obtained for doxorubicin, a widely used anthracycline antibiotic with multiple mechanisms of action. Doxorubicin causes double-strand breaks in the target DNA, so it may be particularly effective for the cells lacking error-free repair of this type of lesion. Some investigations demonstrated high sensitivity of *BRCA*-deficient cells to doxorubicin [24,45,46], while other reports described entirely opposite findings [26, 33, 34. 36]. Another topoisomerase II inhibitor, etoposide, showed selective efficacy against *BRCA*-defective cell in all but one studies [23-25,36,47,48].

Analysis of microtubule poisons produced even more complicated picture. It has been repeatedly demonstrated that *BRCA1*-deficient cells are significantly less sensitive to taxanes or vinca alkaloids than cells with preserved *BRCA1 *function [23,25,26,49-51]. Although these observations are in good agreement with the established role of *BRCA1 *in cellular response to microtubule damage [20], one cannot ignore the existence of sound contradictory data. Zhou et al. [52] reported increased sensitivity of *BRCA1*-mutated ovarian cancer (OC) cell line to paclitaxel as compared to isogenic cells with reconstituted *BRCA1 *function. Tassone et al. [53] showed high sensitivity of *BRCA1*-deficient breast cancer cells to vinorelbine and argued that the differences in the mechanism of action between various microtubule interfering drugs have to be considered while interpreting the results of *BRCA1 *studies. DeLigio and Zorio [20] commented that the tissue origin of the *BRCA1*-mutated cells may be essential in determining the response to taxanes and vinca alkaloids. *BRCA2 *preclinical studies suggested little impact of the status this gene in determining the response to microtubule interfering agents [27,33,46,47].

Alkylating agents are almost always included in the standard schemes for the treatment of breast and ovarian cancers. Surprisingly, this class of drugs has not been subjected to systematic studies in *BRCA*-deficient model systems. Single-agent cyclophosphamide showed only slight antitumor activity against *BRCA1*-mutated human breast cancer xenografts growing in nude mice [31]. At the same time, high-throughput pharmaceutical screen involving *BRCA2*-deficent vs. *BRCA2*-proficient mouse mammary tumor cell lines identified alkylating agents (chlorambucil, melphalane, nimustine) as the most potent and specific inhibitors of cell growth; furthermore, high efficacy of these drugs was confirmed in animal experiments [38].

Topoisomerase I inhibitors are rarely used for the treatment of breast cancer, but included in some therapeutic schemes for ovarian cancer. High sensitivity to these drugs was suggested for both *BRCA1*- [24,54] and *BRCA2*-defective cells [38,46,55], although controversial results have been reported as well [27].

There is a good agreement in the literature that single-agent antimetabolites, 5-fluorouracil and gemcitabine, do not exert specific action against *BRCA*-deficient tumors [24,33,46]. In contrast, 6-thioguanine was identified by a chemical library screen as the most potent antagonist of *BRCA2*-mutated cells [46].

There is a growing number of studies demonstrating pronounced efficacy of specific inhibitors of poly (ADP-ribose) polymerase (PARP) against *BRCA*-deficient cancers [31,33,38-40,56-60]. It is suggested that inactivation of PARP interferes with the repair of spontaneous DNA single-strand breaks. In the normal cells these lesions are converted to double-strand breaks during DNA replication and then eliminated by homologous recombination. Since hereditary cancers are deficient for HR due to loss of both alleles of either *BRCA1 *or *BRCA2*, they cannot eliminate double-strand breaks by error-free mechanism. As result, cancers arising in *BRCA *carriers are selectively sensitive to PARP inhibitors, while the normal tissues from the same individuals retain a non-affected *BRCA *allele and are therefore capable to compensate the consequences of decreased PARP activity [61,62]. PARP inhibitors appear to be the only class of drugs which was assessed in preclinical models in combination with other anticancer agents; synergism of PARP with platinum compounds and alkylating agents has been reported [31,33,38,39,59].

#### Breast cancer

The majority of *BRCA2*- and a certain fraction of *BRCA1*-related BC express estrogen and/or progesterone receptors and are therefore expected to benefit from endocrine therapy. While a couple of studies examined the chemopreventive impact of tamoxifen or oophorectomy in *BRCA *carriers [63] and some investigators analyzed benefits from the adjuvant use of estrogen antagonists [64], there is no reports assessing the role of *BRCA *status in determining the effect of endocrine intervention in neoadjuvant or metastatic setting.

Data on the efficacy of conventional chemotherapeutic schemes in *BRCA*-related vs. sporadic breast cancers are summarized in the Table 2. Several research groups reported outcomes of anthracycline-based therapy. Delagoge et al. [65], Chappuis et al. [66], Warner et al. [67], Hubert et al. [68], Fourquet et al. [69] and Byrski et al. [70] provided evidence for remarkable sensitivity of *BRCA1*/2-related cancers to the neoadjuvant anthracycline-containing regimens. Interestingly, Hubert et al. [68] and Wong Wong Keet et al. [71] observed worse outcomes in *BRCA2 *vs. *BRCA1 *carriers. The data of Petit et al. [72] are in strong disagreement with the above observations: in their study only 2/12 (17%) of *BRCA1 *carriers receiving 5-fluorouracil, epirubicin and cyclophosphamide achieved pathologic complete response (pCR), while pCR was detected in 21/43 (49%) sporadic triple-negative BC.

The only available study of metastatic BC included patients treated by either anthracycline-based or CMF (CMF-like) therapy [73]. In contrast to neoadjuvant series of Hubert et al. [68] and Wong Wong Keet et al. [71], significantly improved outcomes were detected in *BRCA2 *but not *BRCA1 *carriers. Low efficacy of CMF therapy in *BRCA1*-related BC was also described by Byrski et al. [70].

Several investigators analyzed the use of taxane-containing schemes. Byrski et al. [70] observed low rate of pCR in patients with *BRCA1*-mutated BC receiving neoadjuvant combination of doxorubicin and docetaxel. Moiseyenko et al. [74] reported lack of response of chemonaive triple negative *BRCA1*-linked BC to the epirubicin-docetaxel doublet. Wysocki et al. [75] genotyped 19 non-responders to docetaxel and revealed as many as 5 (26%) *BRCA1 *carriers. Kriege et al. [76] confirmed poor efficacy of docetaxel in *BRCA1 *carriers, while *BRCA2*-related BC did not fare worse as compared to sporadic controls. It appears, that the preclinical and clinical evidence warning against the early use of taxanes for the treatment of *BRCA1*-related subtype of BC has already achieved a critical threshold; probably, specifically designed retrospective studies assessing *BRCA1 *status in distinct categories of taxane users may accelerate further understanding of this issue [77].

The experience of the use of single-agent cisplatin for the treatment of hereditary BC is still limited to *BRCA1 *carriers. Byrski et al. [70] reported 10/12 (83%) pCR in patients undergoing neoadjuvant treatment. Silver et al. [78] used cisplatin for the preoperative treatment of triple-negative BC; both *BRCA1 *carriers included in the study demonstrated pCR. Moiseyenko et al. [74] observed major response to cisplatin in a patient whose metastatic BC was insensitive to the upfront anthracycline-taxane combination. The development of resistance to cisplatin may involve the appearance of secondary mutations, which restore *BRCA1 *reading frame and therefore function of the corresponding protein [61,79]. One may expect that the newly acquired *BRCA1 *proficiency will result in sensitization of the cancer cells to estrogen antagonists and taxanes [74].

There are a few case reports on the extremely successful use of high dose chemotherapy in metastatic *BRCA*-related BC. Since *BRCA*-deficient BC are particularly sensitive to DNA damaging agents, use of intensive cytotoxic treatment may provide additional benefit to this category of patients. Furthermore, high dose therapy is likely to counteract tumor adaptation to the drugs, e.g. to induce rapid killing of cancer cells and therefore decrease the probability of developing secondary *BRCA1*-restoring mutations. It is also important to consider that *BRCA*-driven cancers are often characterized by young age at onset, i.e. the majority of these patients would retain sufficient health conditions to qualify for a risky medical intervention. Huang et al. [80] described a patient with metastatic *BRCA2*-related BC, who was treated by high dose chemotherapy and remains disease-free for more than 11 years. Vollebergh et al. [81] presented long term outcomes for 40 metastatic BC patients treated by high dose chemotherapy. 6 patients remained on complete remission for 56+ - 150+ months, and all these 6 patients demonstrated chromosomal imbalances characteristic for *BRCA1*-related cancers. Complete long term responders included 1 out of 2 *BRCA1*- and 1 out of 2 *BRCA2*-carriers.

The only prospective clinical trial specifically designed for *BRCA *carriers evaluated the efficacy of the PARP inhibitor olaparib (AZD2281, KU0059436) [82]. The study included metastatic breast cancer patients, who progressed on the standard chemotherapy schemes. When olaparib was given 400 mg twice daily, objective response and disease stabilization were observed in 11/27 (41%) and 12/27 (44%) patients, respectively. Median progression-free survival approached to 5.7 months.

In agreement with preclinical findings, cisplatin and olaparib clearly outperform conventional treatment schemes when administered to *BRCA1*-driven BC cases. However, both these drugs have limited duration of response, so their use may require the addition of other anticancer agents [31,33,38,39,59].

#### Ovarian cancer

*BRCA*-deficiency in cancer cell can be caused either by germ-line mutation followed by the "second hit", or by somatic inactivation of the *BRCA1 *gene. *BRCA*-inactive tumors constitute the minority of breast cancers (up to 10-15%), and are usually accumulated among family history positive or triple-negative cases. In contrast to BC, the majority of ovarian carcinomas have signs of *BRCA *inactivation, commonly defined in the literature as "*BRCA*ness" [9,15]. Frequent *BRCA*-deficiency in OC appears to be a plausible explanation of the clinical success of platinum-based schemes in the treatment of this disease.

Three studies compared response to the standard chemotherapeutic regimens in *BRCA1*/2- mutated vs. sporadic OC cases [83-85]. These reports provide consistent evidence for higher sensitivity of *BRCA*-driven OC to platinum-containing treatments as compared to the mutation-negative tumors. Interestingly, prolonged tumor responses were documented both for taxane-free schemes and for the combination of platinating drugs with paclitaxel [83-86].

Two independent large trials evaluated the efficacy of olaparib in *BRCA*-mutated OC patients, who experienced prior chemotherapy [87,88]. Audeh et al. [87] observed objective response in 33% and stable disease in 36% of women receiving olaparib at dose 400 mg twice daily. Fong et al. [88] reported tumor response in 40% and disease stabilization in 6% patients, respectively; as expected, higher efficacy of olaparib was documented in those cases, which retained sensitivity to platinum-based therapy.

#### Other genes and other tumors

Hereditary BC research led to identification of several genes other than *BRCA1 *and *BRCA2*. *CHEK2 *appears to be the most studied gene of this class. It confers elevated risk of breast cancer, while its heterozygous occurrence among ovarian cancer patients is not elevated [89,90]. *CHEK2*-mutated BC frequently express estrogen receptor [91,92]. Inactivation of *CHEK2 *by RNA-interference increased cell sensitivity to PARP inhibition [93]. The only available clinical observation describes BC progression in 2 out of 3 *CHEK2 *carriers, who were treated by neoadjuvant single-agent epirubicin, while this outcome was rare (8/104, 8%) in the non-carriers [94].

*PALB2 *(Partner And Localizers of *BRCA2*) has been proven to cause breast and pancreatic hereditary cancer [95,96]. In agreement with *BRCA2*-related function of the *PALB2*, pancreatic cancer xenografts obtained from a *PALB2 *carrier demonstrated pronounced sensitivity to cisplatin and mitomycin C [97]. Importantly, excellent concordance between in vitro and clinical data was observed for this patient: his poorly differentiated ductal adenocarcinoma of the pancreas failed standard gemcitabine therapy, but demonstrated durable tumor response after mitomycin C or cisplatin administration [97]. Increased drug sensitivity of pancreatic tumors obtained from *BRCA2 *carriers was described in several other case reports [98-100]. Therefore, hereditary pancreatic cancers have clearly more favorable pattern of drug response as compared to sporadic cases. Similarly, excellent treatment effect lasting for more than 10 years was documented for *BRCA2*-related advanced lung cancer, which was treated by mitomycin C, cisplatin, and vincristine [101]. Another *BRCA2 *carrier, who suffered from castration-resistant prostate cancer, showed durable marker response and resolution of bone metastases after the administration of olaparib [102]. Vesprini et al. [103] have described a case of metastatic *BRCA2*-related prostate cancer, which was treated by cisplatin after becoming insensitive to androgen ablation. This therapy resulted in normalization of prostate-specific antigen level and symptomatic relief for period of 8 months; docetaxel was administered after the disease progression, and also led to an evident tumor response.

Sokolenko et al. [104] have recently revealed a role of *BLM *gene mutations in hereditary predisposition to breast cancer. This study included 5 patients treated by conventional neoadjuvant therapy; nearly complete pathological response was observed in 3 cases, while the remaining 2 women showed partial reduction of the tumor mass.

Preclinical data suggest specific drug sensitivity pattern for the cells with inactivated *NBN (NBS1) *and *BRIP1 (FANCJ, BACH1) *genes [29,93]. It may turn to be difficult to validate these findings in the clinical setting, due to rarity and population-specific distribution of mutations in the mentioned genes.

### Hereditary non-polyposis colorectal cancer

Hereditary non-polyposis colorectal cancer (HNPCC) is caused by germ-line mutations in *MLH1, MSH2, PMS2 *and *MSH6 *genes. Virtually all tumors from HNPCC mutation carriers are characterized by the defect of mismatch repair (MMR), which is manifested by so-called high-level microsatellite instability (MSI-H). MSI-H occurs in up to 15% of colorectal cancers (CRC), however the majority of the microsatellite-unstable carcinomas are sporadic; hereditary CRC constitute approximately one fifth of MSI-H cases and account for only 2-3% of the total CRC incidence. Mutations in HNPCC-related genes may also predispose to a number of non-colonic tumors, including endometrial, gastric, urothelial, ovarian and some other neoplasms [105,106].

Given the rarity of hereditary CRC and the requirement of expensive multigene test for its definite diagnosis, the collection of clinical series for this disease represents a challenge. Instead, there is an intensive research focusing on MSI-H tumors as a distinct CRC entity; although sporadic and hereditary MSI-H CRC tumors share essential bioclinical features, many experts warn against combined analysis of these two tumor subsets. It is emphasized, that while hereditary CRC affect relatively young subjects, sporadic MSI-H cases are accumulated among elderly patients. Hereditary CRC arise due to mutational inactivation of the *MLH1, MSH2*, *PMS2 *or *MSH6*; sporadic MSI-H tumors are usually driven by methylation of the *MLH1 *gene promoter that may be a consequence of wide-spread abnormalities of epigenetic regulation ("methylator phenotype"). For unknown reason, *BRAF *mutations occur only in sporadic but not in hereditary MSI-H tumors [106,107].

Although MSI-H tumors tend to be poorly differentiated, they are usually characterized by favorable disease course. In particular, MSI-H tumors show relatively low relapse rates after potentially curative surgery [108,109]; in accordance with this, only 4% of advanced CRC have MSI-H phenotype [110]. As result, MSI-H cases are exceptionally rare in trials involving metastatic CRC, therefore the direct clinical assessment of their chemosensitivity is highly complicated. The majority of treatment response data for MSI-H cases is derived from the adjuvant trials, where the reliable discrimination between prognostic and predictive significance of a given parameter is not always possible. Another critical issue concerns technical aspects of determination of microsatellite instability. The existing approaches for detection of MSI-H phenotype are not fully standardized and may be a subject of significant interlaboratory variations [111]. In particular, there is a debate concerning the inclusion of dinucleotide microsatellite markers in the "Bethesda panel", which is frequently used for MSI-H diagnosis [112]. Many opinion leaders insist, that only mononucleotide markers (e.g., *BAT26*) allow to differentiate between true MSI-H and irrelevant mutational noise; hence, consideration of dinucleotide loci may increase the frequency of false-positive MSI-H detection and further compromise the conclusions of clinical trials [111].

Sensitivity of MMR-deficient cells to various anticancer drugs has been a subject of multiple laboratory studies. It is important to acknowledge, that naturally occurring MSI-H cancer cells have highly increased mutation rate and therefore accumulate significant number of "secondary" genetic lesions; depending of the spectrum of the target genes, these secondary lesions may substantially modify the response to treatment modalities [110,113]. Furthermore, inactivation of distinct MMR genes, e.g. *MSH2 *and *MLH1*, may result in distinct patterns of chemosensitivity [114].

Most of preclinical studies point at resistance of MSI-H cells to 5-fluorouracil (5-FU) [110,115]. MSI-H status is also associated with low sensitivity to cisplatin, carboplatin, 6-thioguanine, however these compounds are anyway not engaged in routine CRC treatment [110,115-117]. While MMR-deficiency is associated with non-response to cisplatin and carboplatin, the third platinating drug, oxaliplatin, does not require functional MMR for its action [118]. Several studies have demonstrated specific sensitivity of MSI-H cells to irinotecan; it has been shown, that the response to topoisomerase I poisons may be mediated by the presence of secondary mutations in the double strand break response genes MRE11 and RAD50 [113]. Screen of the library of cytotoxic drugs has identified methotrexate as selective inhibitor of MSH2-deficient cells; MLH1-defective cells did not show specific sensitivity to this compound [114]. Martin et al. [119,120] have recently identified PINK1 kinase and several DNA polymerases as potential targets in cells with mutated MMR genes.

There is a good consensus in the literature that MSI-H CRC patients do not benefit from 5-fluorouracil based adjuvant therapy [121]. Some reports have suggested even worse outcome in treated vs. non-treated patients; it is speculated that ineffective adjuvant therapy may compromise natural immune response to MSI-H cells [110,122,123]. One of the adjuvant patient series specifically included hereditary CRC cases, and also demonstrated lack of benefit from 5-fluorouracil [124]. Given an improved prognosis of MSI-H tumors, it is generally agreed that adjuvant therapy should be omitted for the stage II microsatellite unstable CRC [110,125,126]. Combination of 5-fluorouracil with oxaliplatin has been recently incorporated in the guidelines for adjuvant treatment of stage III CRC; as only a few MSI-H patients with follow-up are currently available, it is impossible to draw conclusions from the existing data sets [127,128]. Trials with irinotecan did not qualify this drug for the use in adjuvant setting; however, the analysis of subset of patients with MSI-H has demonstrated, that this specific category of CRC patients may benefit from addition of irinotecan to fluorouracil and leucovorin [129].

Data on the use of chemotherapy for advanced MSI-H CRC are limited by a few small studies. Liang et al. [130] and Brueckl et al. [131] reported improved response of microsatellite unstable CRC to the 5-fluorouracil-based therapy. There is conflicting information regarding the role of MSI status in determining response to the combination of 5-fluorouracil and oxaliplatin combination [132-134]. Several reports suggested increased response rate of MSI-H CRC to irinotecan [135-137], although this statement was disputed by the recent study of Kim et al. [138].

Burn et al. [139] analyzed the effect of aspirin and resistant starch, given either alone or in combination, on the occurrence of colorectal neoplasia in the *MLH1, MSH2 *or *MSH6 *mutation carriers. Despite encouraging preclinical and epidemiological evidence, neither of these compounds influenced the risk of adenoma formation during the four years of the study.

#### Familial adenomatous polyposis

Familial adenomatous polyposis (FAP) is manifested by multiple polyps, which cause severe gastrointestinal symptoms and frequently progress into cancer lesions. Classical FAP is caused by a dominant germ-line mutation of the *APC *gene. Some patients bear an attenuated form of this disease; mild manifestation of FAP may indicate the involvement of another genetic lesion, i.e. homozygous inactivation of *MUTYH *gene [140]. Development of colonic adenomas usually involves activation of cyclooxygenase 2. Clinical trial involving the specific inhibitor of this enzyme, celecoxib (Onsenal), demonstrated 28% reduction of the number of polyps and 30.7% reduction of the sum of polyp diameters in patients receiving this drug at 400 mg twice daily for 6 months [141]. Based on the results of this trial, celecoxib has been approved for the treatment of FAP. However the safety of its long term-use is questioned by reports revealing elevated rate of cardiovascular events in patients receiving therapeutically effective dose of the drug [142]. Earlier studies demonstrated beneficial effect of sulindac, a non-steroidal anti-inflammatory drug; the results of these trials may be revisited, given that the main adverse effect of this drug, i.e. gastrointestinal toxicity, is medically manageable [143].

#### Hereditary medullary thyroid cancer

Hereditary medullary thyroid cancer (MTC) is caused by germ-line mutation in *RET *tyrosine kinase. It can be a part of multiple endocrine neoplasia (MEN) type 2A (MEN2A) or type 2B (MEN2B) syndromes, or manifest as a single-organ lesion (familial medullary thyroid cancer, FMTC) [144]. A novel multitargeted tyrosine kinase inhibitor vandetanib (ZD6474, Zactima) demonstrates specific activity against mutated RET and inhibits growth of *RET*-transformed cancer cells [145]. A clinical trial involving 30 patients with hereditary MTC, who received 300 mg vandetanib daily, demonstrated objective tumor response in 6/30 (20%) and disease stabilization for more than 24 weeks in 16/30 (53%) cases, respectively [146]. Precise measurement of the change of tumor size revealed the reduction of the lesions in 25/30 (83%) patients; the estimated median progression-free survival approached to 27.9 months [146]. Comparable results were obtained in another trial, which utilized 100 mg daily dosage of this drug [147].

#### Sporadic phenocopies of hereditary cancers

Hereditary neoplasms make relatively little contribution in the total cancer incidence. Nevertheless, advances in the treatment of this category of tumors may have broader practical implications, as many sporadic tumors develop phenotype similar to hereditary cancers. This issue was particularly intensively discussed in breast cancer research, owing to substantial overlap between *BRCA1*-related and triple-negative BC [14]. Given that *BRCA1 *may be inactivated not only by germ-line but also by somatic alterations, several investigations suggested to use *BRCA1 *expression as predictive marker of response to platinum-based and taxane-based therapy [78,148-152]. Other approaches are based on the detection of consequences of either *BRCA *deficiency or other critical defects of homologous recombination; in particular, it has been observed that tumors with presumably impaired repair of DNA double-strand breaks show characteristic pattern of acquired mutations [153,154]. Similarly to *BRCA1*, the mutations of *RET *oncogene are observed not only in hereditary, but also in sporadic medullary thyroid carcinomas; it is expected, that at least a subset of *RET*-driven non-hereditary MTC should respond to vandetanib therapy [144]. While for some tumor types clinical experience is translated from familial cancers to their phenocopies, the reverse flow is observed in colorectal cancer research; as already mentioned above, virtually all data on drug response have been obtained not on a genuine hereditary CRC, but on its phenocopy, i.e. MSI-H tumors; this limitation has to be considered by medical oncologists [106,107].

#### Conclusions and perspectives

Patients with hereditary tumors often benefit from distinct drugs as compared to sporadic cases. The detection of cancer-predisposing germ-line mutations among the participants of clinical trials has rarely been considered, due to significant cost of genetic testing. Given the rapidly increasing accessibility of DNA analysis, it is foreseen that a large number of germ-line mutation carriers will be included in forthcoming trials and/or identified within retrospective collections of biological material. The analysis of correlations between genotype and drug response may substantially improve treatment outcomes, both for hereditary cancer patients and for subjects bearing phenocopies of familial tumors.

## Abbreviations

AC: doxorubicin and cyclophosphamide; BC: breast cancer; CMF: cyclophosphamide, methotrexate and fluorouracil; cCR: clinical complete response; CR: complete response; CRC: colorectal cancer; FAC: 5-fluorouracil, doxorubicin and cyclophosphamide; FAP: familial adenomatous polyposis; FEC: 5-fluorouracil, epirubicin and cyclophosphamide; HNPCC: hereditary non-polyposis colorectal cancer; HR: homologous recombination; MMR: mismatch repair; MTC: medullary thyroid cancer; MSI-H: high-level microsatellite instability; OC: ovarian cancer; OR: objective response; OS: overall survival; PARP: poly (ADP-ribose) polymerase; pCR: pathological complete response; PD: progressive disease; PFS: progression-free survival; PR: partial response; SD: stable disease.

## Competing interests

The authors declare that they have no competing interests.

## Authors' contributions

Both authors contributed to the literature search, data analysis and manuscript preparation. Both authors read and approved the final manuscript.
